# Comparing the efficacy of different antibiotic regimens on osteomyelitis: A network meta-analysis of animal studies

**DOI:** 10.3389/fmed.2022.975666

**Published:** 2022-10-06

**Authors:** Xiangwen Shi, Yipeng Wu, Haonan Ni, Xi Chen, Yongqing Xu

**Affiliations:** ^1^Graduate School, Kunming Medical University, Kunming, China; ^2^Department of Orthopedic Surgery, 920th Hospital of Joint Logistics Support Force, Kunming, China; ^3^School of Health, Brooks College, Sunnyvale, CA, United States; ^4^Department of Epidemiology and Statistics, School of Public Health, Medical College, Zhejiang University, Hangzhou, China

**Keywords:** osteomyelitis, antibiotic regimens, bacteria, network meta-analysis, animal

## Abstract

**Background:**

Despite the surge in the number of antibiotics used to treat preclinical osteomyelitis (OM), their efficacy remains inadequately assessed.

**Objective:**

To establish network comparisons on the efficacy of antibiotic regimens on OM in animal studies.

**Methods:**

PubMed, Embase, Web of Science, and The Cochrane Library were searched from inception to March 2022 for relevant articles. Odds ratios (ORs) were generated for dichotomous variants, and the standard mean difference (SMD) was calculated for constant variables. The predominant outcomes were the effective rate of sterility, also known as sterility rates, as well as the bacterial counts at the end of the experiments and antibiotic concentrations in serum or bone. All the network meta-analyses were performed using STATA MP 16.0. This study was registered in the International Prospective Register of Systematic Reviews (PROSPERO; no. CRD42022316544).

**Results:**

A total of 28 eligible studies with 1,488 animals were included for data analysis, including 13 antibiotic regimens. Regarding the effective rate of sterility, glycopeptides (GLY), linezolid (LIN), rifampicin (RIF)+β-Lactam, and β-Lactam showed significant efficacy compared with placebo (OR ranging from 0.01 to 0.08). For radiological grade, only RIF+GLY (SMD: −5.92, 95%CI: −11.65 to −0.19) showed significant efficacy compared with placebo. As for reducing bacteria count, fosfomycin (FOS), tigecycline (TIG), GLY, LIN, RIF, RIF+β-Lactam, RIF+GLY, aminoglycosides (AMI), and clindamycin (CLI) showed significant efficacy compared with placebo (SMD ranging from −6.32 to −2.62). Moreover, the bone concentrations of GLY were higher 1 h after administration and the higher blood concentrations were higher after 1 h and 4 h compared with the other antibiotics.

**Conclusion:**

Multiple antibiotic regimens showed significant efficacy in animals with OM, including increasing effective rates of sterility, reducing bacterial counts, and lowering radiological scores. Among them, RIF+GLY was the most promising treatment regimen owing to its optimal efficacy. Based on the preclinical studies included in our meta-analysis, head-to-head clinical randomized controlled trials are required to confirm these findings in humans.

## Introduction

Osteomyelitis (OM) is a disease of progressive bone destruction caused by infectious microorganisms and is an intractable complication observed following orthopedic surgery, trauma, or arthroplasty (1). Staphylococcus aureus (*S. aureus*) is the most common disease-causing pathogen, accounting for 65–80% of the microbes found in patients (2). With more advanced detection means, the diagnostic rate of OM has increased worldwide in recent years. Notwithstanding, the mortality rates of adult spinal OM was remain as high as 6% in developed countries (3).

In general, the standard treatment procedures for OM include thorough surgical debridement and systemic antibiotic administration. Although surgical treatment is indispensable, the antibiotics used to fight infections and inhibit biofilm should be chosen carefully because they can have a significant impact on the outcomes.

Currently, for OM with *S. aureus* including Methicillin-resistant *S. aureus* (MRSA) and Methicillin-sensitive *S. aureus* (MSSA) infection, long-term use of sensitive glycopeptides (GLY) antibiotics such as vancomycin and teicoplanin is recommended (1). In addition to the classical antibiotic regimens mentioned above, broad-spectrum antibiotics are used as a monotherapy or as an adjuvant medication for the treatment of OM, such as cephalosporins, aminoglycosides (AMI), penicillins, carbapenems, sulfonamides, and quinolones (QUI) with satisfactory efficacy. The combination of antibiotics for the treatment of OM has become a research hotspot in recent years. For instance, rifampicin (RIF) in combination with QUI has been shown to be effective in implant-associated OM in several studies, given its good permeability and bioavailability (4, 5). Other combination regimens, including RIF combined with GLY or β-Lactam antibiotics, have also been shown to achieve satisfactory results (6, 7).

With so many regimen combinations to use, surgeons face the issue of being overwhelmed with the specific regimen choice. Hence, direct comparisons are required to quantify efficacy. However, after performing a thorough search of the literature in both Chinese and English databases, it was found that the number of studies was lower than the number of interventions possible, thus it was determined that the literature on clinical trials was inadequate for performing network meta-analysis. Consequently, we adopted animal studies to compare efficacy instead: A network meta-analysis on the effects of antibiotics on OM animal models was, therefore, performed. A total of 13 antibiotic regimens were compared: RIF, tigecycline (TIG), linezolid (LIN), fosfomycin (FOS), azithromycin (AZI), clindamycin (CLI), trimethoprim (TRI), GLY, AMI, β-Lactam, QUI, RIF+β-Lactam, and RIF+GLY.

## Materials and methods

This study was performed in accordance with the PRISMA guidelines (see Supplementary Table 1) and the protocol for this meta-analysis can be found on the PROSPERO website (www.crd.york.ac.uk/PROSPERO/; no. CRD42022316544).

### Search strategies

PubMed, Embase, Web of Science, and The Cochrane library were searched from inception to March 2022 for relevant articles using the corresponding Medical Subject Headings (MeSH) “osteomyelitis,” “chronic osteomyelitis,” “anti-bacterial agents,” “antibiotic agent,” “animal,” and “animal experimentation” with Boolean modifiers as appropriate. The in-depth search strategy for the four electronic databases is shown in Supplementary Table 2.

### Inclusion criteria

1. All subjects were OM animal models with no limits on species, age, sex, or weight;
2. The antibiotic regimens included any type of antibiotic regimen or combination of two antibiotics;
3. The study included a negative control group;
4. The outcomes compared the effective rates of sterility, bacterial counts, radiological grades, and antibiotic concentrations in serum or bone.

### Exclusion criteria

1. Clinical research and non-controlled studies using animals;
2. Secondary literature (e.g., literature comment or review);
3. Studies limited to *in vitro* experiments;
4. Studies assessing interventions other than antibiotics (e.g., antibiotic carriers or scaffolds).

### Study selection and data extraction

All identified studies were independently screened by two researchers (X. Shi and Y. Wu) based on the titles and abstracts. Subsequently, they prudently and independently performed the extracted data based on the pre-established data extraction checklist. The extracted data were finally collated into a table and included: (1) basic characteristics of eligibility studies, including the author's name, year of publication, and region of study; (2) basic information on experimental animals, weights, sample sizes, modeling methods, interventions, total therapy time, and other adverse events; (3) outcome indicators: effective rates of sterility, bacterial counts, radiological scores of bone, and antibiotic concentrations in serum or bone. The means and standard deviations (SD) or standard error means (SEM) were extracted from both the antibiotic regimens and control groups as outcome indicators. Any discrepancy was adjudicated by a senior investigator (Y. Xu).

### Assessment of methodological quality

The Systematic Review Center for Laboratory animal Experimentation Risk of Bias (SYRCLE's RoB) tool for animal studies was utilized by two well-trained investigators (X. Shi and Y. Wu) independently to assess the quality of enrolled studies (8). Any discrepancy was adjudicated by a senior investigator (Y. Xu). This SYRCLE's RoB tool is based on the Cochrane Collaboration risk of bias tool (9) and has been adjusted for the risk of bias assessment in animal studies with a total of 10 entries related to six types of bias (selection bias, performance bias, attrition bias, follow-up bias, reporting bias, and other biases). Moreover, specific evaluation criteria were as follows: a “yes” judgment to the assessment question indicates a low risk of bias and a “no” indicated a high risk of bias. When there were insufficient details based on reports to properly assess the risk of bias, “unclear” was assigned to indicate this.

### Outcome indicators

The primary outcomes were the effective rates of sterility (sterility rates after treatment), radiological grades, and bacteria counts.

The secondary outcomes were antibiotic concentrations in serum or bone.

### Statistical analysis

Odds ratios (ORs) were generated for dichotomous variants, while the standard mean difference (SMD) was calculated for constant variables regardless of the type of meta-analysis. *P* < 0.05 was considered statistically significant with a 95% confidence interval (CI). For network meta-analysis, the node analysis model was used to check for inconsistencies. *P* < 0.05 indicated that there was an inconsistency between direct comparison and indirect comparison. Surface under the cumulative ranking curve (SUCRA) values were calculated and matrices were generated; a higher value for the former indicated a higher possibility of being the best treatment. Random effect models were used for network meta-analysis. Moreover, matrices were implemented to detect whether the difference between any one pair with corresponding SUCRAs reached significance. To reinforce the results, overall and loops inconsistency tests and consistency tests were performed in each outcome, and the small sample effect was explored using a network funnel plot. All the network meta-analyses were performed using STATA MP 16.0.

## Results

### Study characteristics and quality assessment

**Figure 1:**
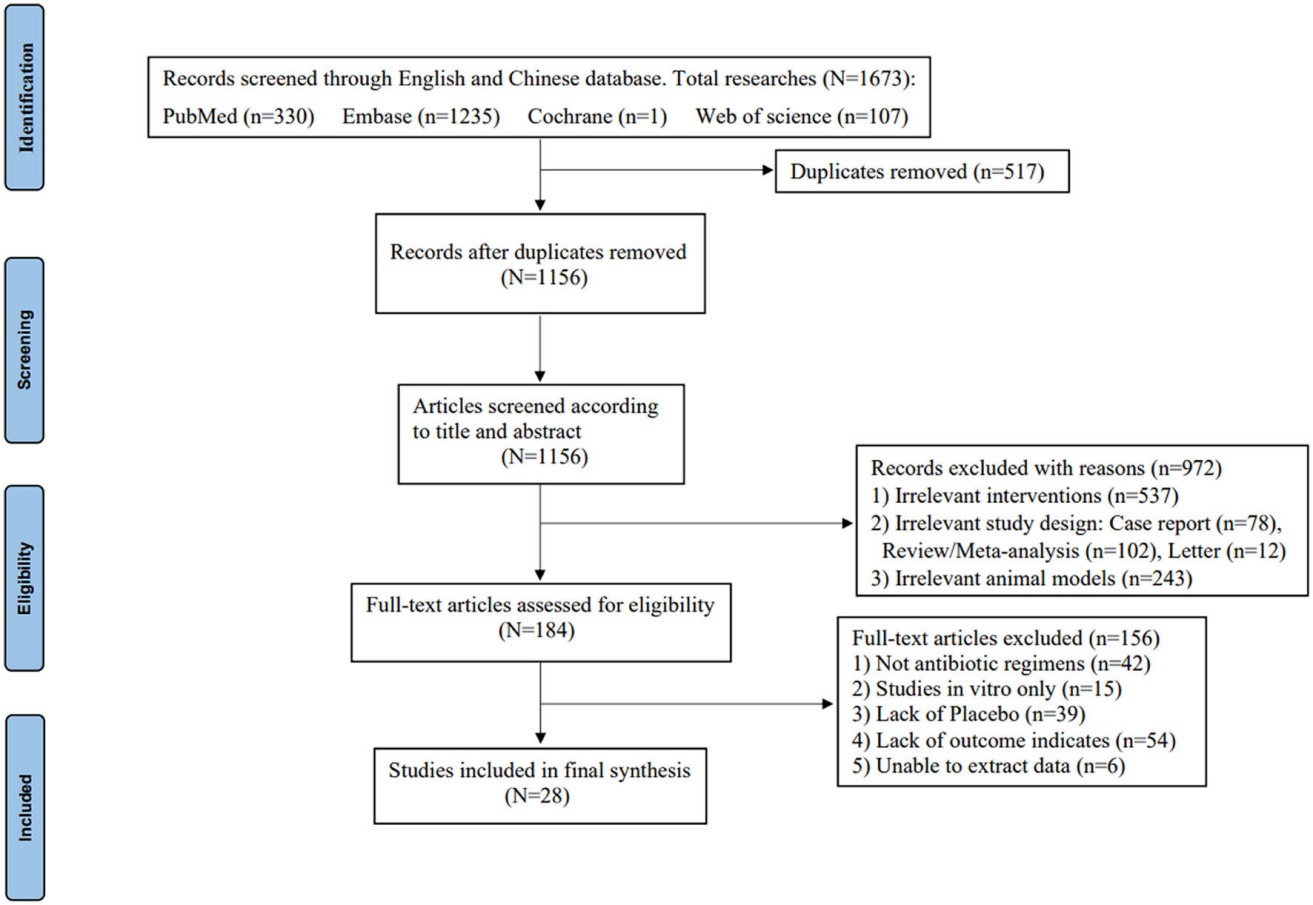
PRISMA flow diagram of the study selection process (10).

In total, 28 controlled studies consisting of a total of 1488 animals were included, and a total of 13 antibiotic regimens were used. The flow diagram of the selection criteria is shown in Figure 1. Of the included studies, experimental animal species included *New Zealand White rabbits* (11–23), *Sprague-Dawley (SD) rats* (24–29), *Wistar rats* (30–35), *Madorin rats* (36, 37), or *RAR rats* (38). The animal infection models included *S. aureus, MRSA, Methicillin-resistant Staphylococcus epidermidis (MRSE), Klebsiella pneumoniae, or Morganella*-infected animals. The methods of OM induction in six studies were implant-based models, and the remaining studies were all post-traumatic models. The duration of administration ranged from 4 to 28 days. In addition, antibiotic regimens were administrated intraperitoneally, subcutaneously, intravenously, intramuscularly, or orally during the treatment phase. The basic characteristics of the animal studies included are shown in Supplementary Table S1.

**Figure 2:**
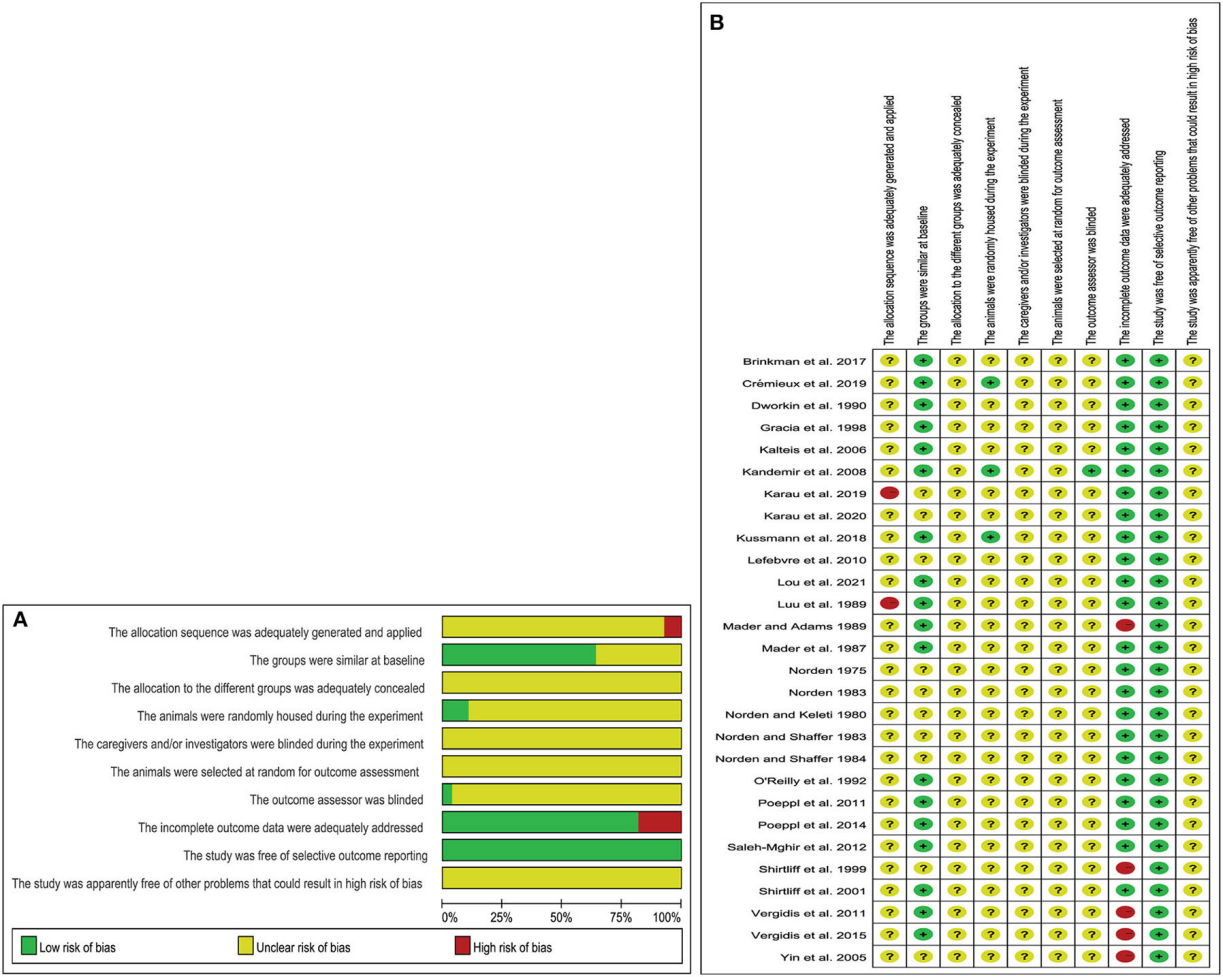
Risk of bias **(A)** graph showing the risk of bias; **(B)** individual risk of bias for each of the included animal studies.

Of the 28 controlled studies included, no studies reported detailed randomization grouping methods and generation processes; 18 studies reported similar baseline characteristics of animals; three studies described in detail that experimental animals were placed in a randomized housing environment. Only one study described a blinded assessment of outcomes. In five studies, animal deaths were reported after antibiotic treatment. All studies reported expected results. The results of the methodological quality assessment are shown in Figures 2A,B.

### Effective rates of sterility

**Figure 3:**
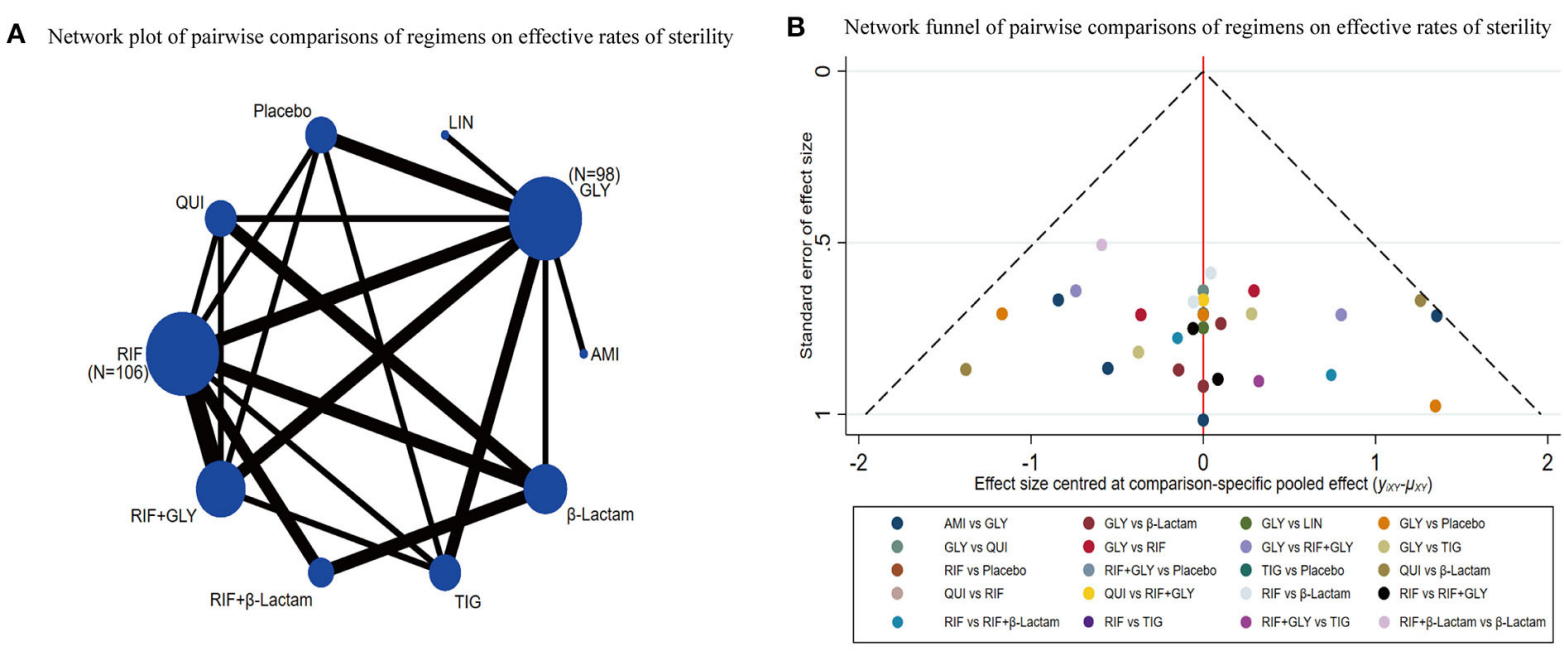
Network meta-analysis of the total effective rates of sterility **(A)** network graph; **(B)** the network funnel plot.

A total of 12 out of 28 studies were included for this endpoint. Network graphs of each pairwise comparison of all regimens on effective rates of sterility are shown in Figure 3A. RIF and GLY had the highest number of studies. From the matrix (Table 1), out of 10 interventions, only GLY, LIN, RIF+β-Lactam, and β-Lactam showed significant efficacy compared with the placebo, whereas the rest were inefficient. Additionally, RIF+β-Lactam was equivalent to GLY, LIN, and β-Lactam. The network funnel plot suggested that the small sample effect existed in the comparison between AMI and GLY on total effective rates of sterility (Figure 3B).

**Table 1:** Matrix of pairwise comparations of regimens on effective rates of sterility [shown as odds ratios (ORs) and 95% confidence intervals (CIs)].

	**Placebo**	**AMI**	**QUI**	**GLY**	**TIG**	**RIF**	**RIF+GLY**	**LIN**	**β-Lactam**	**RIF+β-Lactam**
RIF+β-Lactam	**0.01 (0.00,0.31)**	0.47 (0.02,10.96)	0.08 (0.00,1.85)	**0.12 (0.01,1.46)**	0.12 (0.01,1.46)	0.16 (0.03,0.69)	0.16 (0.01,1.88)	**0.23 (0.01,5.84)**	**0.34 (0.01,10.03)**	1
β-Lactam	**0.03 (0.00,0.79)**	0.15 (0.01,2.93)	0.24 (0.01,4.69)	0.36 (0.04,3.55)	0.36 (0.02,7.45)	0.46 (0.02,9.59)	0.46 (0.02,9.60)	0.69 (0.03,14.88)	1	2.96 (0.10,87.58)
LIN	**0.04 (0.00,0.97)**	0.31 (0.01,7.57)	0.35 (0.02,5.64)	0.52 (0.07,4.01)	0.52 (0.03,9.00)	0.67 (0.04,11.59)	0.67 (0.04,11.60)	1	1.45 (0.07,31.48)	4.30 (0.17,108.00)
RIF+GLY	0.06 (0.00,1.41)	1.93 (0.12,30.22)	0.52 (0.03,8.13)	0.78 (0.11,5.71)	0.78 (0.11,5.71)	1.00 (0.14,7.36)	1	1.50 (0.09,26.02)	2.18 (0.10,45.55)	6.44 (0.53,78.12)
RIF	0.06 (0.00,1.41)	0.90 (0.04,19.94)	0.52 (0.03,8.13)	0.78 (0.11,5.71)	0.78 (0.11,5.71)	1	1.00 (0.14,7.37)	1.50 (0.09,26.02)	2.18 (0.10,45.55)	6.44 (1.44,28.82)
TIG	0.08 (0.00,1.81)	0.21 (0.01,3.36)	0.67 (0.04,10.43)	1.00 (0.14,7.31)	1	1.29 (0.18,9.43)	1.29 (0.18,9.43)	1.93 (0.11,33.37)	2.80 (0.13,58.41)	8.28 (0.68,100.13)
GLY	**0.08 (0.01,0.88)**	0.60 (0.05,6.94)	0.67 (0.10,4.45)	1	1.00 (0.14,7.30)	1.28 (0.18,9.42)	1.29 (0.18,9.42)	1.92 (0.25,14.87)	2.80 (0.28,27.82)	8.27 (0.68,100.03)
QUI	0.12 (0.01,2.56)	7.44 (0.24,226.61)	1	1.50 (0.22,10.02)	1.50 (0.10,23.46)	1.93 (0.12,30.22)	1.93 (0.12,30.24)	2.89 (0.18,47.06)	4.20 (0.21,82.69)	12.41 (0.54,284.98)
AMI	12.37 (0.55,276.12)	1	0.13 (0.00,4.09)	1.66 (0.14,19.18)	4.67 (0.30,73.23)	1.11 (0.05,24.48)	0.52 (0.03,8.14)	3.20 (0.13,77.52)	6.75 (0.34,133.29)	2.14 (0.09,49.96)
Placebo	1	0.08 (0.00,1.81)	8.25 (0.39,174.01)	12.37 (1.14,134.44)	12.37 (0.55,276.12)	15.89 (0.71,355.63)	15.90 (0.71,355.89)	23.81 (1.03,551.35)	34.64 (1.26,949.66)	102.37 (3.25,3225.08)

### Radiological grades

**Figure 4:**
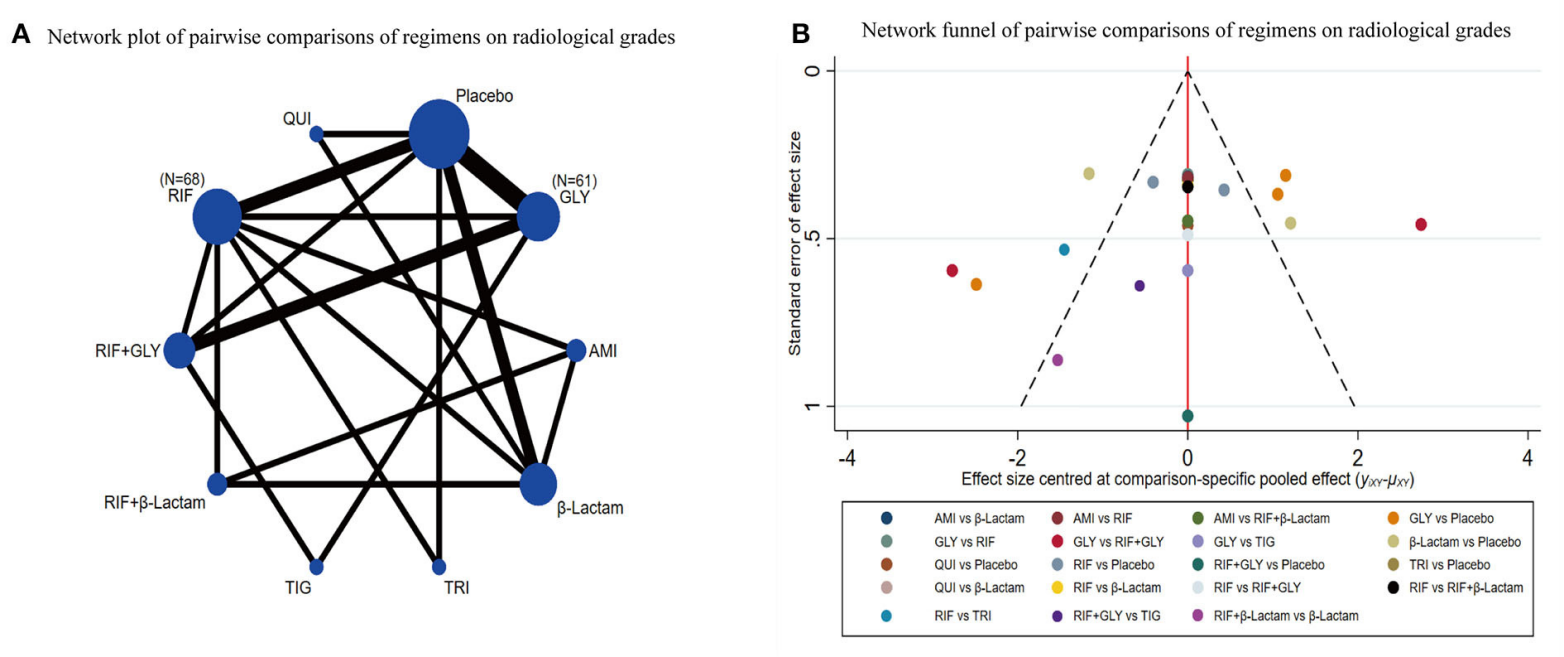
Network meta-analysis of radiological grades **(A)** network graph; **(B)** the network funnel plot.

Of the 28 studies, eight were included for this endpoint. A network graph of each of the pairwise comparisons of all the regimens on effective rates of sterility is shown in Figure 4A. RIF and GLY had the highest number of studies. According to the matrix (Table 2), out of 10 interventions, only RIF+GLY showed significant efficacy compared to placebo, while the others were ineffective. The network funnel plot suggested that the small sample effect existed in the comparison between RIF+GLY and the placebo on radiological grades (Figure 4B).

**Table 2:** Matrix of pairwise comparations of regimens on radiological grades [shown as SMD (Standardized mean difference) and 95% confidence intervals (CIs)].

	**Placebo**	**TRI**	**TIG**	**β-Lactam**	**GLY**	**QUI**	**AMI**	**RIF+β-Lactam**	**RIF**	**RIF+GLY**
RIF+GLY	**5.92 (0.19,11.65)**	6.04 (−0.61,12.70)	5.44 (−1.94,12.81)	3.67 (−2.91,10.25)	3.37 (−3.20,9.95)	3.37 (−4.01,10.76)	−2.33 (−9.68,5.02)	2.78 (−3.79,9.36)	2.78 (−1.88,7.45)	1
RIF	3.14 (−2.56,8.84)	3.26 (−3.37,9.89)	2.65 (−4.70,10.00)	0.88 (−3.77,5.53)	0.59 (−4.06,5.23)	0.59 (−6.77,7.95)	−0.03 (−5.76,5.69)	0.00 (−4.64,4.64)	1	−2.78 (−7.45,1.88)
RIF+β-Lactam	3.14 (−4.21,10.48)	3.26 (−4.83,11.34)	2.65 (−6.03,11.34)	0.88 (−3.77,5.53)	0.59 (−4.06,5.24)	0.59 (−8.11,9.29)	0.56 (−4.08,5.19)	1	−0.00 (−4.64,4.64)	−2.78 (−9.36,3.79)
AMI	−1.65 (−7.36,4.05)	−3.34 (−8.01,1.33)	6.04 (−0.61,12.70)	−0.55 (−7.10,5.99)	0.03 (−6.52,6.58)	2.58 (−0.74,5.90)	1	−0.56 (−5.19,4.08)	0.03 (−5.69,5.76)	2.33 (−5.02,9.68)
QUI	2.55 (−2.12,7.22)	2.67 (−4.76,10.10)	2.06 (−4.52,8.65)	0.29 (−8.41,8.99)	−0.00 (−8.70,8.70)	1	−2.58 (−5.90,0.74)	−0.59 (−9.29,8.11)	−0.59 (−7.95,6.77)	−3.37 (−10.76,4.01)
GLY	2.55 (−4.79,9.89)	2.67 (−5.41,10.76)	2.06 (−6.62,10.75)	0.29 (−4.36,4.94)	1	0.00 (−8.70,8.70)	−0.03 (−6.58,6.52)	−0.59 (−5.24,4.06)	−0.59 (−5.23,4.06)	−3.37 (−9.95,3.20)
β-Lactam	2.26 (−5.09,9.60)	2.38 (−5.71,10.47)	1.77 (−6.92,10.46)	1	−0.29 (−4.94,4.36)	−0.29 (−8.99,8.41)	0.55 (−5.99,7.10)	−0.88 (−5.53,3.77)	−0.88 (−5.53,3.77)	−3.67 (−10.25,2.91)
TIG	0.49 (−4.16,5.13)	0.61 (−6.81,8.02)	1	−1.77 (−10.46,6.92)	−2.06 (−10.75,6.62)	−2.06 (−8.65,4.52)	−6.04 (−12.70,0.61)	−2.65 (−11.34,6.03)	−2.65 (−10.00,4.70)	−5.44 (−12.81,1.94)
TRI	−0.12 (−5.91,5.67)	1	−0.61 (−8.02,6.81)	−2.38 (−10.47,5.71)	−2.67 (−10.76,5.41)	−2.67 (−10.10,4.76)	3.34 (−1.33,8.01)	−3.26 (−11.34,4.83)	−3.26 (−9.89,3.37)	−6.04 (−12.70,0.61)
Placebo	1	0.12 (−5.67,5.91)	−0.49 (−5.13,4.16)	−2.26 (−9.60,5.09)	−2.55 (−9.89,4.79)	−2.55 (−7.22,2.12)	1.65 (−4.05,7.36)	−3.14 (−10.48,4.21)	−3.14 (−8.84,2.56)	−5.92 (−11.65,−0.19)

### Bacterial counts

**Figure 5:**
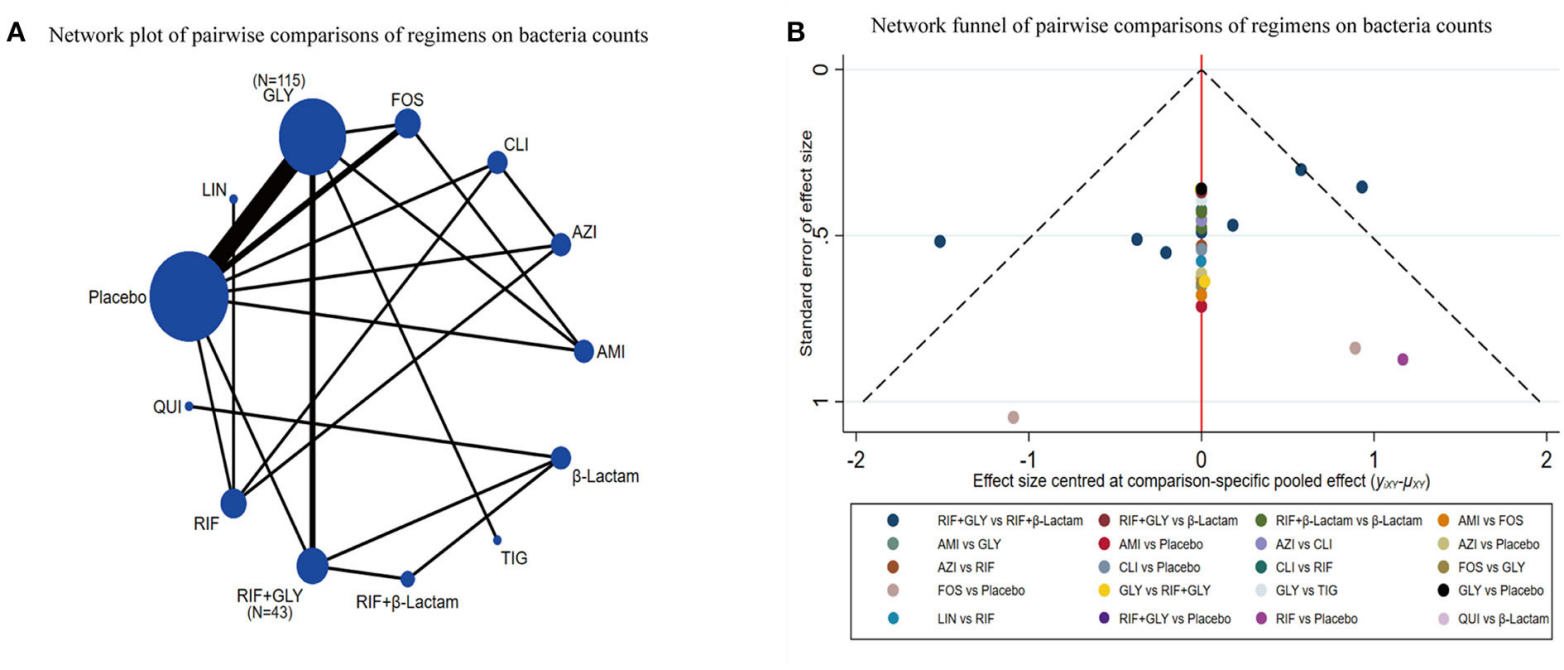
Network meta-analysis of bacteria counts **(A)** network graph; **(B)** the network funnel plot.

Of the 28 studies, 14 were included for this endpoint. A network graph of each of the pairwise comparisons of all regimens on bacterial counts is shown in Figure 5A. GLY and RIF+GLY had the highest number of studies. From the matrix (Table 3), nine of the 13 interventions showed significant efficacy compared with the placebo, including AMI, CLI, FOS, GLY, LIN, RIF, RIF+GLY, RIF+β-Lactam, and TIG. AZI was significantly inferior to FOS, GLY, LIN, RIF, and TIG. FOS was significantly superior to AMI, AZI, CLI, QUI, RIF+GLY, and β-Lactam. AMI was significantly superior to RIF+β-Lactam and LIN. The network funnel plot suggested that the small sample effect existed in the comparison between FOS and the placebo on bacteria counts (Figure 5B).

**Table 3:** Matrix of pairwise comparations of regimens on bacterial counts [shown as SMD (Standardized mean difference) and 95% confidence intervals (CIs)].

	**Placebo**	**QUI**	**AZI**	**β-Lactam**	**CLI**	**AMI**	**RIF+GLY**	**RIF+β-Lactam**	**RIF**	**LIN**	**TIG**	**GLY**	**FOS**
FOS	**6.32 (3.83,8.81)**	**5.60 (1.47,9.73)**	**5.19 (2.07,8.32)**	**4.14 (0.46,7.83)**	**3.70 (0.52,6.88)**	**3.32 (1.22,5.42)**	**3.27 (0.14,6.40)**	2.98 (−0.70,6.66)	2.63 (−0.58,5.83)	2.30 (−1.42,6.01)	1.16 (−2.41,4.72)	1.24 (−1.77,4.25)	1
GLY	**5.08 (2.10,8.06)**	4.36 (−0.09,8.80)	**3.95 (0.43,7.48)**	2.90 (−1.13,6.93)	2.46 (−1.12,6.03)	−2.07 (−5.76,1.62)	2.03 (−1.50,5.55)	1.73 (−2.29,5.76)	1.38 (−2.21,4.98)	1.06 (−3.00,5.11)	−0.09 (−1.99,1.81)	1	−1.24 (−4.25,1.77)
TIG	**5.17 (1.64,8.70)**	4.44 (−0.39,9.28)	**4.04 (0.03,8.04)**	2.99 (−1.47,7.44)	2.54 (−1.51,6.59)	−2.28 (−6.16,1.61)	2.11 (−1.89,6.12)	1.82 (−2.63,6.27)	1.47 (−2.59,5.54)	1.14 (−3.34,5.62)	1	0.09 (−1.81,1.99)	−1.16 (−4.72,2.41)
LIN	**4.02 (1.26,6.78)**	3.30 (−1.00,7.61)	**2.90 (0.19,5.61)**	1.85 (−2.03,5.72)	1.40 (−1.29,4.09)	**−3.61 (−6.75,−0.48)**	0.97 (−2.38,4.32)	0.68 (−3.19,4.55)	0.33 (−1.55,2.21)	1	−1.14 (−5.62,3.34)	−1.06 (−5.11,3.00)	−2.30 (−6.01,1.42)
RIF	**3.70 (1.68,5.71)**	2.97 (−0.90,6.85)	**2.57 (0.62,4.52)**	1.52 (−1.87,4.91)	1.07 (−0.85,3.00)	1.02 (−2.41,4.46)	0.64 (−2.13,3.41)	0.35 (−3.03,3.73)	1	−0.33 (−2.21,1.55)	−1.47 (−5.54,2.59)	−1.38 (−4.98,2.21)	−2.63 (−5.83,0.58)
RIF+β-Lactam	**3.35 (0.63,6.06)**	2.62 (−0.10,5.35)	2.22 (−1.09,5.53)	1.17 (−0.81,3.14)	0.72 (−2.64,4.08)	**−3.00 (−5.05,−0.95)**	0.29 (−1.65,2.23)	1	−0.35 (−3.73,3.03)	−0.68 (−4.55,3.19)	−1.82 (−6.27,2.63)	−1.73 (−5.76,2.29)	−2.98 (−6.66,0.70)
RIF+GLY	**3.05 (1.15,4.95)**	2.33 (−0.38,5.04)	1.93 (−0.76,4.61)	0.87 (−1.08,2.83)	0.43 (−2.32,3.18)	1.76 (−1.62,5.15)	1	−0.29 (−2.23,1.65)	−0.64 (−3.41,2.13)	−0.97 (−4.32,2.38)	−2.11 (−6.12,1.89)	−2.03 (−5.55,1.50)	−3.27 (−6.40,−0.14)
AMI	**4.47 (0.97,7.96)**	−2.08 (−4.24,0.08)	1.87 (−0.92,4.66)	−0.70 (−3.57,2.18)	0.38 (−2.48,3.23)	1	−1.76 (−5.15,1.62)	3.00 (0.95,5.05)	−1.02 (−4.46,2.41)	3.61 (0.48,6.75)	2.28 (−1.61,6.16)	2.07 (−1.62,5.76)	−3.32 (−5.42,−1.22)
CLI	**2.62 (0.64,4.61)**	1.90 (−1.95,5.76)	1.50 (−0.44,3.43)	0.45 (−2.92,3.81)	1	−0.38 (−3.23,2.48)	−0.43 (−3.18,2.32)	−0.72 (−4.08,2.64)	−1.07 (−3.00,0.85)	−1.40 (−4.09,1.29)	−2.54 (−6.59,1.51)	−2.46 (−6.03,1.12)	−3.70 (−6.88,−0.52)
β-Lactam	2.18 (−0.54,4.90)	1.46 (−0.42,3.33)	1.05 (−2.27,4.37)	1	−0.45 (−3.81,2.92)	0.70 (−2.18,3.57)	−0.87 (−2.83,1.08)	−1.17 (−3.14,0.81)	−1.52 (−4.91,1.87)	−1.85 (−5.72,2.03)	−2.99 (−7.44,1.47)	−2.90 (−6.93,1.13)	−4.14 (−7.83,−0.46)
AZI	1.13 (−0.77,3.02)	0.41 (−3.40,4.21)	1	−1.05 (−4.37,2.27)	−1.50 (−3.43,0.44)	−1.87 (−4.66,0.92)	−1.93 (−4.61,0.76)	−2.22 (−5.53,1.09)	−2.57 (−4.52,−0.62)	−2.90 (−5.61,−0.19)	−4.04 (−8.04,−0.03)	−3.95 (−7.48,−0.43)	−5.19 (−8.32,−2.07)
QUI	0.72 (−2.58,4.03)	1	−0.41 (−4.21,3.40)	−1.46 (−3.33,0.42)	−1.90 (−5.76,1.95)	2.08 (−0.08,4.24)	−2.33 (−5.04,0.38)	−2.62 (−5.35,0.10)	−2.97 (−6.85,0.90)	−3.30 (−7.61,1.00)	−4.44 (−9.28,0.39)	−4.36 (−8.80,0.09)	−5.60 (−9.73,−1.47)
Placebo	1	−0.72 (−4.03,2.58)	−1.13 (−3.02,0.77)	−2.18 (−4.90,0.54)	−2.62 (−4.61,−0.64)	−4.47 (−7.96,−0.97)	−3.05 (−4.95,−1.15)	−3.35 (−6.06,−0.63)	−3.70 (−5.71,−1.68)	−4.02 (−6.78,−1.26)	−5.17 (−8.70,−1.64)	−5.08 (−8.06,−2.10)	−6.32 (−8.81,−3.83)

### Antibiotic concentrations in the serum

Antibiotic concentrations in serum 1 hour after administration (μg/ml): a network graph of each pairwise comparison of all the regimens is shown in Supplementary Figure S1A. GLY and AMI had the highest number of studies. From the matrix (Supplementary Table S2), RIF was significantly lower to GLY (SMD: −6.51, 95%CI: −9.28 to −3.75), AMI (SMD: −3.56, 95%CI: −5.27 to −1.84), and β-Lactam (SMD: −3.48, 95%CI: −5.20 to −1.75). The network funnel plot suggested that the small sample effect existed in the comparison between GLY and RIF on the antibiotic concentration of serum 1 h after administration (Supplementary Figure S1B).

Antibiotic concentrations in the serum 4 h after administration (μg/ml): a network graph of each pairwise comparison of all the regimens is shown in Supplementary Figure S2A. RIF and GLY had the highest number of studies. From the matrix (Supplementary Table S3), GLY was significantly higher than RIF (SMD: 3.20, 95%CI: 0.52 to 5.89), AMI (SMD: 5.90, 95%CI: 2.46 to 9.17), TRI (SMD: 5.80, 95%CI: 2.01 to 9.58), and β-Lactam (SMD: 6.64, 95%CI: 3.36 to 9.92). The network funnel plot suggested that the small sample effect existed in the comparison between RIF and β-Lactam on the antibiotic concentration of serum after 4 h after administration (Supplementary Figure S2B).

### Antibiotic concentrations in bone

Antibiotic concentrations in the bone 1 h after administration (μg/g): a network graph of each pairwise comparison of all the regimens is shown in Supplementary Figure S3A. GLY and AMI had the highest number of studies. From the matrix (Supplementary Table S4), GLY was significantly higher than β-Lactam (SMD: 4.40, 95%CI: 0.32 to 8.47), RIF (SMD: 5.28, 95%CI: 1.89 to 8.67), and TRI (SMD: 6.28, 95%CI: 1.62 to 10.95). The network funnel plot suggested that the small sample effect existed in the comparison between GLY and RIF on the antibiotic concentration of bone after 1 h after administration (Supplementary Figure S3B).

Antibiotic concentrations in the bone 4 h after administration (μg/g): a network graph of each pairwise comparison of all the regimens is shown in Supplementary Figure S4A. RIF and GLY had the highest number of studies. From the matrix (Supplementary Table S5), we conducted pairwise comparisons among five antibiotic regimens. However, there was no significant difference between these regimens. The network funnel plot suggested that the small sample effect existed in the comparison between RIF and TRI on the antibiotic concentrations in the bone 4 h after administration (Supplementary Figure S4B).

## Discussion

To the best of our knowledge, this is the first network meta-analysis comparing the efficacy of antibiotic regimens on OM in preclinical animal models. We found that the combination of RIF+GLY was more effective than the placebo in the analysis of radiological grades and bacteria counts after treatment and a similar animal study on OM came to the same conclusion (39). There was only one previous meta-analysis with antibiotics for OM (40), and their analysis showed no difference in the efficacy of quinolones and β-Lactams in treating patients with OM, results similar to those obtained in our network meta-analysis.

Interestingly, we analyzed blood and bone concentrations after antibiotic administration and found that GLY bone concentrations were higher 1 h after administration and blood concentrations were higher 1 and 4 h after administration compared with the other antibiotics. Current guidelines recommend serum vancomycin concentrations of 15–20 μg/ml in adult patients with *S. aureus* infection in the clinic (41), and our analysis described serum concentrations of GLY in OM models, in which the mean serum concentration at 4 h was 18.7 ± 5 μg/ml, approaching the guideline recommendations. However, due to the significant difference in metabolic rates between rats and humans (42, 43), we should be very careful in extrapolating results from animal models to humans. Additionally, the local concentration of antibiotics in the tissue must exceed the MIC of the bacteria to eradicate the infection (44). In our included studies, MIC-matched 18–24 h blood and bone concentrations were not provided, and therefore the exact efficacy of the drug could not be assessed.

The causative agents of OM include several types of bacteria, including Gram-positive, Gram-negative, and multi-species mixed infections. In general, the antibiotic must cross the outer membrane and cytoplasmic membrane of the bacteria before entering the cytoplasm to exert its anti-bacterial effects. More importantly, the aggregation of surface-associated microorganisms forms biofilms, and the penetration specificity of antibiotics is directly related to the efficacy of OM (45, 46). In addition to biofilm formation, both Gram-negative and Gram-positive bacteria can actively expel antibiotics from cells *via* efflux pumps, which is another major drug resistance mechanism (47). Gram-negative bacteria are usually more resistant to the action of anti-bacterial drugs than Gram-positive bacteria due to the presence of porins (48, 49).

Rifampicin is a broad-spectrum antimicrobial agent with penetration specificity for biofilms produced by *S. aureus* and it can kill adherent bacteria (50). Because of the powerful biofilm penetration capacity, RIF is used as a basic therapeutic measure in the clinical treatment of OM. Nevertheless, the use of RIF alone in the treatment of *S. aureus*-associated infections is likely to rapidly develop drug resistance, increase MIC, and reduce the effectiveness of antibiotics (51, 52). Therefore, the antibiotic regimen of RIF combined with drugs has been used in preclinical and clinical studies for nearly two decades. GLY antibiotics lyse bacteria by binding to their cell wall peptidoglycans and primarily target most Gram-positive bacteria; however, they have significant limitations, such as poor tissue and intracellular permeability, lack of activity against biofilms, and a slow bactericidal effect (53). More importantly, RIF enhances the activity of vancomycin against *S. aureus* in biofilms (54), compensating for the above disadvantages of GLY. In terms of clinical efficacy, GLY antibiotics such as vancomycin remain first-line agents for the treatment of *S. aureus* or MRSA-induced OM. Recently, a case of successfully treated polymicrobial calcaneal OM using oral RIF in combination with intravenous vancomycin was described (55).

The combination of RIF and β-Lactam is a common pairing in the clinical treatment of bone infections. β-Lactam antibiotics inhibit the synthesis of bacterial cell walls and affect normal bacterial growth and development (56, 57). Among β-Lactams, cefuroxime and cephalothin have been shown to be effective in the presence of biofilm (58). However, β-Lactams are the most common cause of most antibiotics-associated adverse events, including severe kidney or liver toxicity, neurotoxicity, and cytopenia (59). One study showed a 2.5% incidence of gastrointestinal events with β-Lactam and a higher incidence of diarrhea when the above combination was used (60). More importantly, the β-Lactams assessed in this study included only first and second-generation cephalosporins, and no valid direct evidence could be produced to support the efficacy of the novel cephalosporins on OM.

Fosfomycin is a broad-spectrum antibiotic that inhibits the biosynthesis of peptidoglycan in the bacterial cell wall by inhibiting MurA enzyme activity and it exhibits bactericidal activity against a wide range of Gram-negative bacteria (61, 62). Although FOS demonstrated a significant advantage in reducing bacterial counts compared to RIF+GLY in our analysis, FOS is commonly used in the treatment of complicated urinary tract infections, and its use as a clinical treatment for OM has not been reported. Importantly, the incidence of hypokalemia with intravenous FOS infusion was as high as 26%, a very high incredible rate (63). In contrast, the nephrotoxicity of vancomycin could be avoided by reducing the dose of the drug or by replacing it with teicoplanin, which exhibits less nephrotoxicity. The primary adverse reactions of RIF were gastrointestinal reactions and certain hepatotoxic effects (transaminase levels increased to 3–5 times normal levels). Perveen et al. (64), Ibrahim et al. (65); however, the gastrointestinal symptoms and hepatotoxicity of RIF could also be addressed by lowering the dose (66).

In terms of cost, the median daily drug price of RIF for infection was less than 0.115 dollars (67). For the treatment of *S. aureus*-associated infection, the cost of vancomycin was generally very low, while for MRSA the cost increased accordingly. The problem of refractory MRSA-associated OM has been solved with the use of new GLY antibiotics including dalbavancin and teicoplanin, which also have the advantage of shorter hospital stays and low total costs.

Thus, RIF+GLY may be the most promising clinical option for OM treatment in terms of biofilm penetration, clinical feasibility, cost-effectiveness, and safety. However, the drug–drug interactions between RIF and co-antibiotics may reduce the effective blood levels of the co-antibiotics (68). Conversely, it is worth paying attention to the high incidence of side effects of RIF combinations, which highlights the potential need for therapeutic drug monitoring and rational administration during clinical treatment.

### Adverse reaction analysis

In the present study, only a few studies briefly reported adverse reactions in animals after administration, including seven rabbits in the TIG group that died from gastrointestinal inflammation and seven rabbits in the RIF+β-Lactam group that died from gastrointestinal reactions (diarrhea and gastrointestinal inflammation). Moreover, two rabbits and three rats in the RIF+GLY group, and one rabbit in the GLY group had an unknown cause of death. Gastrointestinal inflammation in the TIG and RIF+β-Lactam groups may be caused by extensive destruction of the normal intestinal flora (20). Additionally, gastrointestinal adverse effects of RIF include common symptoms, such as loss of appetite and diarrhea; however, gastrointestinal inflammation is rare (69). Therefore, in future, the adverse effects of antibiotic combinations should be considered in the selection of antibiotic regimens for the treatment of OM.

### Limitations

First, due to a lack of clinical literature and the absence of primary outcomes, we used preclinical animal studies to assess the efficacy of various antibiotic regimens. Nevertheless, we could give clear recommendations based on the existing studies on humans.

Second, the pathogenic bacteria used to establish the OM animal model were not identical and included some uncommon bacteria, such as *Klebsiella pneumoniae* and *Morganella*. After conventional meta-analysis low heterogeneity in conventional meta-analysis for S. aureus and MRSA separately, but this might still affect the interpretation of efficacy.

Third, in our selection of animal model species for OM, we included only the most common rat and rabbit as target models, and therefore the efficacy results should be interpreted with caution. The primary reason for this is that most of the current comparative studies on antibiotic treatments for OM have focused on rat and rabbit models of OM, and other models such as dog, pig, and sheep lack valid comparative evidence.

### Research prospects

First, selection of animal models: Current therapeutic animal models for OM are limited to rats and rabbits, such animals differ greatly from humans *in vivo*, particularly with regard to the pathological molecular biology, thus they have limited reference value regarding clinical treatment (70). Therefore, animal models that are more similar to humans, such as monkeys and apes, should be considered in future to better simulate the pathological conditions of clinical OM.

Second, safety indicators: Only a very small number of studies have reported the gastrointestinal adverse effects of antibiotics, and most studies focused on efficacy and bacterial eradication rates. Therefore, attention should be paid to the safety of antibiotics in future to avoid adverse events in clinical applications. In addition, preclinical trials for OM treatment should take into account the rigor of design and the standardization of outcome assessment to improve the quality of the study and provide a reference for the clinical treatment of OM as much as possible.

Third, as antimicrobial resistance continues to increase worldwide, the biofilm penetration of vancomycin and β-lactam antibiotics for Gram-positive bacteria is diminishing. Novel glycopeptide antibiotics such as oritavancin and telavancin, and fifth-generation cephalosporin antibiotics such as ceftobiprole should be given more attention in future and applied in preclinical and clinical studies to provide a reliable basis for antibiotic treatment options for OM.

Fourth, local antibiotic delivery systems have become the key development direction of preclinical and clinical research for the treatment of OM in recent years (71). In addition to the mechanical support, biocompatibility, biodegradability, and osteoinductive ability of the new material, the combination of new antibiotics also need more research in the animal models of OM to achieve the final clinical application.

Fifth, the vast majority of comparative studies on the efficacy of antibiotics use rabbits and rats as model animals to study OM. However, it has been reported that, in comparison to the serious adverse reactions of rabbits after antibiotic treatment, the gastrointestinal flora of the pig is similar to that of humans and can be used to assess systemic antibiotic treatments (72, 73), and the mechanical and biological functions of dogs' tibia and femur are similar to those of humans (74). Therefore, researchers need to further explore other animal models of OM that are more similar to human bodies and evaluate the efficacy of antibiotics, in order to provide theoretical support for the clinical treatment of OM with antibiotics in future.

## Conclusion

In conclusion, multiple antibiotic regimens have shown significant value in animal models of OM, including increased efficacy, reduced bacterial counts, and lower radiological scores. Among them, RIF + GLY was significantly effective and possibly the most promising treatment regimen. However, it is necessary for future preclinical studies to provide more reliable evidence for the clinical treatment of OM using this regimen.

## Data availability statement

The original contributions presented in the study are included in the article/Supplementary material, further inquiries can be directed to the corresponding authors.

## Author contributions

XS and YX conceived the study and wrote the manuscript. HN, YW, and XC carried out the data collection and data analysis. XS and YW assessed the quality of the studies. All authors reviewed the results and approved the final version of the manuscript.

## Funding

This study was funded by National Natural Science Foundation of China (Grant Nos. 81772367 and 82072392), the Yunnan Traumatology and Orthopedics Clinical Medical Center (Grant No. ZX20191001), and the Grants from Yunnan Orthopedics and Sports Rehabilitation Clinical Medicine Research Center (Grant No. 202102AA310068).

## Conflict of interest

The authors declare that the research was conducted in the absence of any commercial or financial relationships that could be construed as a potential conflict of interest.

## Publisher's note

All claims expressed in this article are solely those of the authors and do not necessarily represent those of their affiliated organizations, or those of the publisher, the editors and the reviewers. Any product that may be evaluated in this article, or claim that may be made by its manufacturer, is not guaranteed or endorsed by the publisher.
