# Supplementary material for: Comparing the efficacy of different antibiotic regimens on osteomyelitis: A network meta-analysis of animal studies
Source: Front Med (Lausanne). 2022 Oct 6;9:975666. doi: 10.3389/fmed.2022.975666 (PMC9582527; doi:10.3389/fmed.2022.975666)
Supplement: Supplementary file 1 [file Data_Sheet_1.pdf]

Supplementary Material

1 Supplementary Data: Search strategies up to March 2022

Search strategy in PubMed:1963-2022.03

#1 osteomyelitis OR osteomyelitides (33196)

#2 "Osteomyelitis"[Mesh] (23952)

#3 #1 OR #2 (33196)

#4 anti-bacterial OR anti bacterial agents OR antibacterial agents OR antibacterial OR antibacterial agent OR anti-bacterial compounds OR anti bacterial compounds OR anti-bacterial agent OR anti bacterial agent OR anti-bacterial compound OR anti bacterial compound OR bacteriocidal agents OR bacteriocidal OR bacteriocidal agent OR bacteriocide OR bacteriocides OR anti-mycobacterial agents OR anti-mycobacterial OR anti mycobacterial agents OR anti-mycobacterial agent OR anti mycobacterial agent OR antimycobacterial agent OR antimycobacterial OR antimycobacterial gents OR antibiotics OR antibiotic (1046786)

#5 "Anti-Bacterial Agents"[Mesh] (809670)

#6 #4 OR #5(1046786)

#7 experimentation, animal OR animal research OR research, animal OR animal experimental use OR animal experimental uses OR experimental use, animal OR

experimental uses, animal OR animal experiments OR animal experiment OR experiment, animal OR experiments, animal (1,060,479)

#8 "Animal Experimentation"[Mesh] (10126)

#9 #7 OR #8 (1060479)

#10 #3 AND #6 AND #9 (330)

Search strategy in Embase:1980-2022.03

#1 ('chronic osteomyelitis':ab,ti,kw or 'experimental osteomyelitis':ab,ti,kw or 'hematogenous osteomyelitis':ab,ti,kw or 'Majeed syndrome':ab,ti,kw or 'Paget bone disease':ab,ti,kw or 'petrositis':ab,ti,kw or 'tuberculous osteomyelitis':ab,ti,kw) (4880)

#2 'osteomyelitis'/exp (48644)

#3 #1 or #2 (49146)

#4 ('antibiotic':ab,ti,kw OR 'antibiotic combination':ab,ti,kw OR 'antibiotic drug':ab,ti,kw OR 'antibiotic ointment':ab,ti,kw OR 'antibiotic residue':ab,ti,kw OR 'antibiotic spectrum':ab,ti,kw OR 'antibiotics':ab,ti,kw OR 'antibiotics and their derivatives':ab,ti,kw OR 'antibiotics, combined':ab,ti,kw OR 'antibiotics, folate antagonists':ab,ti,kw OR 'antibiotics, miscellaneous':ab,ti,kw OR 'antibiotics, nitrofuran':ab,ti,kw OR 'antibiotics, oxalodinones':ab,ti,kw OR 'combined

antibiotic':ab,ti,kw) (525354)

#5 'antibiotic agent'/exp (1749053)

#6 #4 or #5 (1904147)

#7 (animal:de OR 'invertebrate'/exp OR 'amphibia'/exp OR 'fish'/exp OR 'boreoeutheria'/exp OR 'afrotheria'/exp OR 'dermoptera'/exp OR 'glires'/exp OR 'scandentia'/exp OR 'sauropsid'/exp OR 'laurasiatheria'/exp OR 'ungulate'/exp OR 'reptile'/exp OR 'cercopithecidae'/exp OR 'marsupial'/exp OR 'monotremate'/exp OR 'prosimian'/exp OR 'tarsiiform'/exp OR 'hylobatidae'/exp OR 'xenarthra'/exp OR 'platyrrhini'/exp OR 'chimpanzee'/exp OR 'gorilla'/exp OR 'orang utan'/exp OR 'homo neanderthalensis'/exp OR 'cephalochordata'/exp OR 'hyperotreti'/exp OR 'urochordata'/exp OR 'ambulacraria'/exp OR 'coelomata'/exp OR 'protostomia'/exp OR 'pseudocoelomata'/exp OR 'coelenterate'/exp OR 'mesozoa'/exp OR 'placozoa'/exp OR 'porifera'/exp OR 'juvenile animal'/exp OR 'male animal'/exp OR 'female animal'/exp OR 'primate'/de OR 'haplorhini'/de OR 'mammal'/de OR 'catarrhini'/de OR 'simian'/de OR 'ape'/de OR 'amniote'/de OR 'tetrapod'/de OR 'vertebrate'/de OR 'chordata'/de OR 'deuterostomia'/de OR 'bilateria'/de OR 'therian'/de OR 'hominid'/de OR 'euarchontoglires'/de OR 'placental mammals'/de) (7765519)

#8 #3 and #6 AND #7 (1235)

Search strategy in Cochrane library:1980-2022.03

#1 (osteomyelitis OR osteomyelitides):ti,ab,kw (631)

#2 MeSH descriptor: [osteomyelitis] explode all trees (154)

#3 #1 or #2 (638)

#4 (anti-bacterial OR anti bacterial agents OR antibacterial agents OR antibacterial OR antibacterial agent OR anti-bacterial compounds OR anti bacterial compounds OR anti-bacterial agent OR anti bacterial agent OR anti-bacterial compound OR anti bacterial compound OR bacteriocidal agents OR bacteriocidal OR bacteriocidal agent OR bacteriocide OR bacteriocides OR anti-mycobacterial agents OR anti-mycobacterial OR anti mycobacterial agents OR anti-mycobacterial agent OR anti mycobacterial agent OR antimycobacterial agent OR antimycobacterial OR antimycobacterial gents OR antibiotics OR antibiotic):ti,ab,kw (41882)

#5 MeSH descriptor: [Anti-Bacterial Agents] explode all trees (12889)

#6 #4 or #5 (42835)

#7 (experimentation, animal OR animal research OR research, animal OR animal experimental use OR animal experimental uses OR experimental use, animal OR experimental uses, animal OR animal experiments OR animal experiment OR experiment, animal OR experiments, animal):ti,ab,kw (7091)

#8 MeSH descriptor: [Animal Experimentation] explode all trees (3)

#9 #7 or #8 (7092)

#11 #3 and #6 and #9 and #10 (1)

Search strategy in Web of science:1991-2022.03

TS=(osteomyelitis OR osteomyelitides) AND TS=(anti-bacterial agents OR anti-bacterial OR anti bacterial agents OR antibacterial agents OR antibacterial OR antibacterial agent OR anti-bacterial compounds OR anti bacterial compounds OR anti-bacterial agent OR anti bacterial agent OR anti-bacterial compound OR anti bacterial compound OR bacteriocidal agents OR bacteriocidal OR bacteriocidal agent OR bacteriocide OR bacteriocides OR anti-mycobacterial agents OR anti-mycobacterial OR anti mycobacterial agents OR anti-mycobacterial agent OR anti mycobacterial agent OR antimycobacterial agent OR antimycobacterial OR antimycobacterial gents OR antibiotics OR antibiotic) AND TS=(animal experimentation OR experimentation, animal OR animal research OR research, animal OR animal experimental use OR animal experimental uses OR experimental use, animal OR experimental uses, animal OR animal experiments OR animal experiment OR experiment, animal OR experiments, animal) (107)

2 Supplementary Figures and Tables

2.1 Supplementary Tables

Supplementary Table S1. The characteristics of included studies.

First author (country, year)	Species (Sexs)	Model (method)	Weight (g)	Method of OM induction	Intervention	Sample size (n=intervention/control)	Total therapy time (day)	Outcome indicators	Other Adverse Events
**Gracia et al. (Spain, 1998)**	Wistar Rats(male)	2ml S. aureus (ATCC 29213, SP variant;2×10^9CFU/ml) for 5 weeks	average 375	Post-trauma	i.m. β-Lactam(30.0mg/ml) (12000μg; bid) vs i.m. AMI(1.5mg/ml) (600μg; bid) vs i.m. GLY(8.0mg/ml) (3000μg; bid) vs Placebo	28 (8β-Lactam/8AMI/8GLY/4Placebo)	21	1. Effective rate<br>2. CFU in bone<br>3. MIC and MBC	None
**Kandemir et al. (Turkey,2008)**	Sprague-Dawley Rats(female)	0.1ml MRSA (1×10^8CFU/ml) for 2 weeks	160-240	Post-trauma	i.m. TIG (14mg/kg; bid) vs s.c. GLY (20mg/ml; qd) vs Placebo	40 (13TIG/13GLY/14Placebo)	28	1. Effective rate<br>2. CFU in bone<br>3. MIC and MBC	None
**Noden et al. (USA, 1975)**	New Zealand White Rabbits (NR)	By intramedullary injection sodium morrhuate and S. aureus (3×10^6CFU/ml) for 2 weeks	average 1800	Post-trauma	s.c. RIF (40mg/kg; qd) vs s.c. AMI (5mg/kg; bid) vs s.c. β-lactam (50mg/kg; tid) vs	125 (20RIF/20AMI/18β-Lactam/20RIF+β-Lactam/16Placebo)	28	1. Effective rate<br>2. Antibiotic concentrations in serum and bone<br>3. Radiological severity	NR

					RIF+β-Lactam vs Placebo			score<br>4. MIC	
**Saleh-Mghir et al. (France, 2012)**	New Zealand White Rabbits(female)	By intramedullary injection 0.2ml CA-MRSA (LAC;3.4×10^{7}CFU/ml) for 2 weeks	2000-3000	Post-trauma	s.c. β-lactam (40mg/ml; qid) vs s.c. GLY (60mg/ml; bid) vs s.c. RIF (10mg/m; bid)+GLY vs s.c. RIF (10mg/m; bid)+β-Lactam vs Placebo	30 (10β-lactam/11GLY/10RIF+β-lactam/11RIF+GLY/9Placebo)	14	1. Effective rate<br>2. CFU in bone	None
**Luu et al. (USA, 1989)**	RAR Rats(male)	MSSA (52/52A/80) for 3 weeks	average 400	Post-trauma	s.c. GLY (80mg/kg; bid) vs Placebo	33 (17GLY/16Placebo)	14	1. Effective rate<br>2. CFU in bone<br>3. Radiological severity score<br>4. Antibiotic concentrations in serum<br>5. MIC	NR
**Mader and Adams (USA, 1989)**	New Zealand White Rabbits(female)	By intramedullary injection 0.1ml 5% sodium morrhuate and 0.1ml MRSA (1×10^{6}CFU/ml) and 0.2ml sterile saline for 3-4 weeks	1500-2000	Post-trauma	s.c. GLY (40mg/ml; qid) vs Placebo	36 (18GLY/18Placebo)	28	1. Effective rate<br>2. CFU in bone<br>3. Radiological severity score<br>4. Antibiotic concentrations	Three rabbits died after therapy (GLY)

								in serum<br>5. MIC and MBC	
**Poeppl et al. (Austria, 2011)**	Sprague-Dawley Rats(male)	By intramedullary injection 20μl MRSA (4409/07;1×10^{8} to 5×10^{8}CFU/ml) for 4 weeks	350-400	Post-trauma	s.c. GLY (60mg/kg; qd) vs s.c. FOS (75mg/kg; qd) vs Placebo	28 (9GLY/10FOS/9Placebo)	28	1. Effective rate<br>2. CFU in bone<br>3. MIC	NR
**Vergidis et al. (USA, 2014)**	Wistar Rats(male)	By intramedullary injection 50μl MRSA (IDRL-6169;1×10^{6}) for 4 weeks	250-350	Post-trauma	i.p. GLY (50mg/kg; bid) vs i.p. TIG (14mg/kg; bid) vs i.p. RIF (25mg/ml; bid) vs RIF+GLY vs Placebo	80 (16GLY/16TIG/16RIF/16RIF+GLY/16Placebo)	21	1. Effective rate<br>2. CFU in bone<br>3. Antibiotic concentrations in serum<br>4. MIC	Two rabbits died (RIF+GLY) and one fracture
**Noden (USA, 1983)**	New Zealand White Rabbits (NR)	By intramedullary injection sodium morrhuate and S. aureus (3×10^{6}CFU/ml) for 2 weeks	average 1800	Post-trauma	s.c. RIF (40mg/kg; qd) vs s.c. AMI (10mg/kg; bid) vs s.c. β-lactam (50mg/kg; tid) vs s.c. TRI(40mg/kg; qid) vs RIF+β-Lactam vs RIF+GLY vs Placebo	175 (25RIF/25HLAR/25β-lactam/25TRI/25RIF+β-Lactam/25RIF+GLY/25Placebo)	28	1. Effective rate<br>2. Antibiotic concentrations in serum and bone<br>3. Radiological severity score<br>4. MIC	NR

**O'Reilly et al. (USA, 1992)**	Madorin Rats(male)	By intramedullary injection sodium morrhuate and 0.05ml S. aureus(1098;2×10^9CFU/ml) for 10 days	average 200	Post-trauma	s.c. RIF (20mg/kg; qd) vs p.o. AZI (50mg/kg; qd) vs p.o. CLI (90mg/kg; tid) vs Placebo	55 (15RIF/15AZI/10CLI/15Placebo)	21	1. Effective rate<br>2. CFU in bone<br>3. Antibiotic concentrations in serum and bone<br>4. MIC and MBC	NR
**Brinkman et al. (USA,2016)**	Wistar Rats(male)	By intramedullary injection 50μl MRSA(IDRL-6169;1×10^6CFU/ml) for 4 weeks	250-300	Implant	i.p. RIF (25mg/kg; bid)+i.p. GLY (50mg/kg; bid) vs Placebo	32 (16RIF+GLY/16Placebo)	21	1. Effective rate<br>2. CFU in bone<br>3. MIC	NR
**Mader et al. (USA, 1987)**	New Zealand White Rabbits (NR)	By intramedullary injection 0.1ml 5% sodium morrhuate and 0.1ml S. aureus(7×10^6CFU/ml) for 3-4 weeks	average 2000	Post-trauma	s.c. QUI (25mg/kg; bid) vs s.c. β-Lactam (40mg/kg; qid) vs Placebo	60 (20QUI/20β-Lactam/20Placebo)	28	1. Effective rate<br>2. CFU in bone<br>3. Radiological severity score<br>4. Antibiotic concentrations in serum<br>5. MIC and MBC	None
**Kalteis et al. (Germany,2006)**	Wistar Rats(male)	By intramedullary injection 100μl MRSA(ATCC 29213;1×10^8CFU/ml) for one week	average 453	Implant	i.p. QUI (10mg/kg; bid) vs i.p. GLY (15mg/kg; bid) vs Placebo	36 (12QUI/12GLY/12Placebo)	21	CFU in bone	None
**Lefebvre et al. (France,2010)**	New Zealand White Rabbits(female)	By intramedullary injection 1ml MRSA(BCB8;1×10^8CFU/ml) for 3-4 weeks	NR	Implant	i.v. GLY (100mg/kg; qd) vs i.v. RIF (20mg/ml; bid) vs Placebo	30(14GLY/8RIF+GLY/8Placebo)	4	1. Effective rate<br>2. CFU in bone<br>3. Antibiotic concentrations in serum<br>4. MIC	NR

**Shirtliff et al. (USA,1999)**	New Zealand White Rabbits(NR)	By intramedullary injection 0.1ml 5% sodium morrhuate and 0.1ml S. aureus(1×10^7CFU/ml) for 2 weeks	1500-2000	Implant	p.o. RIF (40mg/kg; qd)+s.c. β-Lactam (40mg/kg; qid) vs Placebo	24 (14RIF+β-Lactam/10Placebo)	28	1. Effective rate<br>2. CFU in bone<br>3. Antibiotic concentrations in serum and bone<br>4. MIC and MBC	Seven rabbits died because of excessive dehydration and gastrointestinal inflammation (RIF+β-Lactam)
**Karau MJ(USA,2019)**	Sprague-Dawley Rats(male)	By intramedullary injection 50μl MRSA(IDRL-6169;1×10^7CFU/ml) for one week	NR	Post-trauma	i.p. GLY(60mg/ml; bid) vs Placebo	32 (16GLY/16Placbo)	4	1. CFU in bone	NR
**Poeppl et al. (Austria,2014)**	Sprague-Dawley Rats(male)	By intramedullary injection 15μl MRSA(4409/07/1-5×10^8CFU/ml) for 4 weeks	350-400	Post-trauma	i.p. GLY (50mg/kg; bid) vs i.p. FOS (75mg/kg; qd) vs Placebo	32 (11GLY/10FOS/11Placebo)	28	1. Effective rate<br>2. CFU in bone<br>3. Antibiotic concentrations in bone<br>4. MIC	NR
**Yin et al. (USA,2005)**	New Zealand White Rabbits(NR)	By intramedullary injection 0.15ml 5% sodium morrhuate and 0.1ml MRSA(1×10^6CFU/ml) for 2 weeks	2000-3500	Post-trauma	s.c. TIG (14mg/kg; bid) vs s.c. GLY (30mg/kg; b id) vs p.o. RIF (40mg/kg; bid)+s.c. GLY vs Placebo	46 (10TIG/11GLY/10RIF+GLY/15Placebo)	28	1. Effective rate<br>2. CFU in bone<br>3. Radiological severity score<br>4. Antibiotic concentrations in serum and bone<br>5. MIC and MBC	Seven rabbits died because of gastrointestinal inflammation (TIG); one rabbit died due to intolerance to anesthesia (TIG)

**Noden and Shaffer (USA, 1984)**	New Zealand White Rabbits(NR)	By intramedullary injection sodium morrhuate and Morganella (5×10^7CFU/ml) for 2 weeks	average 1800	Post-trauma	s.c. β-Lactam (400mg/kg; qid) vs Placebo	44 (21β-Lactam/23Placebo)	28	1. Effective rate<br>2. Antibiotic concentrations in serum and bone<br>3. Radiological severity score<br>4. MIC	NR
**Noden and Shaffer (USA, 1983)**	New Zealand White Rabbits(NR)	By intramedullary injection sodium morrhuate and S. aureus(3×10^6CFU/ml) for 2 weeks	average 1800	Post-trauma	s.c. GLY (60mg/kg; bid) vs s.c. RIF(40mg/kg;qd) vs RIF+GLY vs Placebo	86 (22GLY/21RIF/20RIF+GLY/Placebo)	28	1. Effective rate<br>2. Antibiotic concentrations in serum and bone<br>3. Radiographic severity score<br>4. MIC	NR
**Dworkin et al. (USA, 1990)**	Madorin Rats(male)	By intramedullary injection 0.05ml 5% sodium morrhuate and 0.05ml S. aureus(A68) for average 25.6 days	165-200	Post-trauma	s.c. QUI (30mg/kg; bid) vs s.c. GLY (60mg/kg; bid) vs s.c. RIF (20mg/kg; qd) vs RIF+VAN vs Placebo	62 (6QUI/8GLY/6RIF/18RIF+GLY/Placebo)	average 24.2	1. Effective rate<br>2. CFU in bone<br>3. MIC	NR
**Vergidis et al. (USA, 2011)**	Wistar Rats(male)	By intramedullary injection 50μl MRSA(IDRL-6169;1×10^6CFU/ml) for 4 weeks	215-475	Post-trauma	i.p. RIF (25mg/kg; bid) vs i.p. LIN (35mg/ml; bid) vs RIF+GLY (50mg/kg; bid vs Placebo	56 (16RIF/14LIN/13RIF+GLY/13Placebo)	21	1. Effective rate<br>2. CFU in bone<br>3. Antibiotic concentrations in serum<br>4. MIC and MBC	Three rats died (RIF+GLY)

**Kussmann et al. (Austria, 2018)**	Sprague-Dawley Rats(male)	By intramedullary injection 10μl MRSA (40496/08;1-5×10^{6}CFU/ml) for 4 weeks	average 448	Implant	i.p. GLY (50mg/ml; bid) vs placebo	22 (11GLY/11Placebo)	28	1. Effective rate 2. CFU in bone 3. MIC	None
**Noden and Keleti (USA, 1980)**	New Zealand White Rabbits(NR)	By intramedullary injection sodium morrhuate and S. aureus(3×10^{6}CFU/ml) for 2 weeks	average 1800	Post-trauma	s.c. TRI (40mg/kg; qid) vs s.c. RIF (40mg/kg; qd) vs Placebo	60 (20TRI/20RIF/20Placebo)	14	1. Effective rate 2. Antibiotic concentrations in serum and bone 3. Radiological severity score 4. MIC	NR
**Shirtliff et al. (USA,2001)**	New Zealand White Rabbits(female)	By intramedullary injection 0.1ml 5% sodium morrhuate and 0.1ml S. aureus(1×10^{6}CFU/ml) for 2 weeks	1500-3000	Implant	p.o. QUI (30mg/kg; qd) vs s.c. β-Lactam (30mg/kg; qid) vs Placebo	67 (20QUI/20/27Placebo)	28	1. Effective rate 2. CFU in bone 3. MIC and MBC	NR
**Cre´mieux et al. (France,2019)**	New Zealand White Rabbits(female)	By intramedullary injection K. pneumoniae (KPC-99YC;1×10^{9}CFU/ml) for 2 weeks	2000-3000	Post-trauma	i.m. AMI (30mg/kg; qd) vs i.m. TIG (14mg/kg; bid) vs s.c. β-Lactam (80mg/kg; tid) vs i.m. FOS (150mg/kg; bid) vs Placebo	58 (11AMI/11TIG/12β-Lactam/11FOS/13Placebo)	7	1. Effective rate 2. CFU in bone 3. MIC and MBC	None
**Karau et al. (USA,2020)**	Wistar Rats(male)	By intramedullary injection 50μl MRSA(IDRL-	NR	Post-trauma	p.o. RIF (10mg/kg; bid) vs i.p. GLY (100mg/kg; bid) vs	32 (8RIF/8GLY/8RIF+GLY/8Placebo)	15	1. Effective rate 2. CFU in bone	NR

		6169;1×10^{6}CFU/ml) for 4 weeks			RIF+GLY vs Placebo				
**Lou et al. (China,2021)**	Sprague-Dawley Rats(male)	By intramedullary injection 100μl MRSE(1×10^{8}CFU/ml) for one week	250-350	Post-trauma	i.p. LIN (30mg/kg; qd) vs i.p. RIF (25mg/kg; bid) vs i.p. GLY (50mg/kg; bid) vs RIF+GLY vs Placebo	79 (16LIN/15RIF/15GLY/15RIF+GLY/18Placebo)	14	1. Effective rate<br>2. CFU in bone<br>3. MIC and MBC	NR

Supplementary Table S1. Matrix of pairwise comparisons of regimens on antibiotic concentrations in serum at 1h (shown as SMD and 95% confidence intervals)

SUCRA (%)	GLY	AMI	β-Lactam	RIF	TRI
GLY	1	-2.96 (-6.21,0.30)	-3.04 (-6.29,0.22)	**-6.51 (-9.28, -3.75)**	**-6.69 (-10.32, -3.06)**
AMI	2.96 (-0.30,6.21)	1	-0.08 (-1.75,1.59)	**-3.56 (-5.27, -1.84)**	**-3.73 (-6.65, -0.82)**
β-Lactam	3.04 (-0.22,6.29)	0.08 (-1.59,1.75)	1	**-3.48 (-5.20, -1.75)**	**-3.65 (-6.57, -0.74)**
RIF	6.51 (3.75,9.28)	3.56 (1.84,5.27)	3.48 (1.75,5.20)	1	-0.18 (-2.53,2.18)
TRI	6.69 (3.06,10.32)	3.73 (0.82,6.65)	3.65 (0.74,6.57)	0.18 (-2.18,2.53)	1

Supplementary Table S2. Matrix of pairwise comparisons of regimens on antibiotic concentrations in serum at 4h (shown as SMD and 95% confidence intervals)

	GLY	RIF	AMI	TRI	β-Lactam
GLY	1	-3.20 (-5.89, -0.52)	-5.90 (-9.17, -2.64)	-5.80 (-9.58, -2.01)	-6.64 (-9.92, -3.36)
RIF	**3.20 (0.52,5.89)**	1	-2.70 (-4.56, -0.84)	-2.59 (-5.26,0.07)	-3.44 (-5.32, -1.56)
AMI	**5.90 (2.64,9.17)**	**2.70 (0.84,4.56)**	1	0.11 (-3.14,3.36)	-0.74 (-2.58,1.10)
TRI	**5.80 (2.01,9.58)**	2.59 (-0.07,5.26)	-0.11 (-3.36,3.14)	1	-0.85 (-4.11,2.41)
β-Lactam	**6.64 (3.36,9.92)**	**3.44 (1.56,5.32)**	0.74 (-1.10,2.58)	0.85 (-2.41,4.11)	1

Supplementary Table S3. Matrix of pairwise comparisons of regimens on antibiotic concentrations in bone at 1h (shown as SMD and 95% confidence intervals)

	GLY	AMI	β-Lactam	RIF	TRI
GLY	1	-2.92 (-6.99,1.16)	-4.40 (-8.47, -0.32)	-5.28 (-8.67, -1.89)	-6.28 (-10.95, -1.62)
AMI	2.92 (-1.16,6.99)	1	-1.48 (-3.74,0.78)	-2.36 (-4.63, -0.10)	-3.37 (-7.28,0.55)
β-Lactam	**4.40 (0.32,8.47)**	1.48 (-0.78,3.74)	1	-0.88 (-3.14,1.38)	-1.89 (-5.80,2.02)
RIF	**5.28 (1.89,8.67)**	**2.36 (0.10,4.63)**	0.88 (-1.38,3.14)	1	-1.00 (-4.20,2.19)
TRI	**6.28 (1.62,10.95)**	3.37 (-0.55,7.28)	1.89 (-2.02,5.80)	1.00 (-2.19,4.20)	1

Supplementary Table S4. Matrix of pairwise comparisons of regimens on antibiotic concentrations in bone at 4h (shown as SMD and 95% confidence intervals)

	AMI	GLY	RIF	β-Lactam	TRI
AMI	1	0.30 (-6.11,6.70)	-1.09 (-4.79,2.60)	-1.64 (-5.36,2.07)	-4.32 (-10.76,2.12)
GLY	-0.30 (-6.70,6.11)	1	-1.39 (-6.63,3.85)	-1.94 (-8.35,4.48)	-4.62 (-12.06,2.82)
RIF	1.09 (-2.60,4.79)	1.39 (-3.85,6.63)	1	-0.55 (-4.25,3.16)	-3.23 (-8.52,2.06)
β-Lactam	1.64 (-2.07,5.36)	1.94 (-4.48,8.35)	0.55 (-3.16,4.25)	1	-2.68 (-9.14,3.77)
TRI	4.32 (-2.12,10.76)	4.62 (-2.82,12.06)	3.23 (-2.06,8.52)	2.68 (-3.77,9.14)	1

2.2 Supplementary Figures

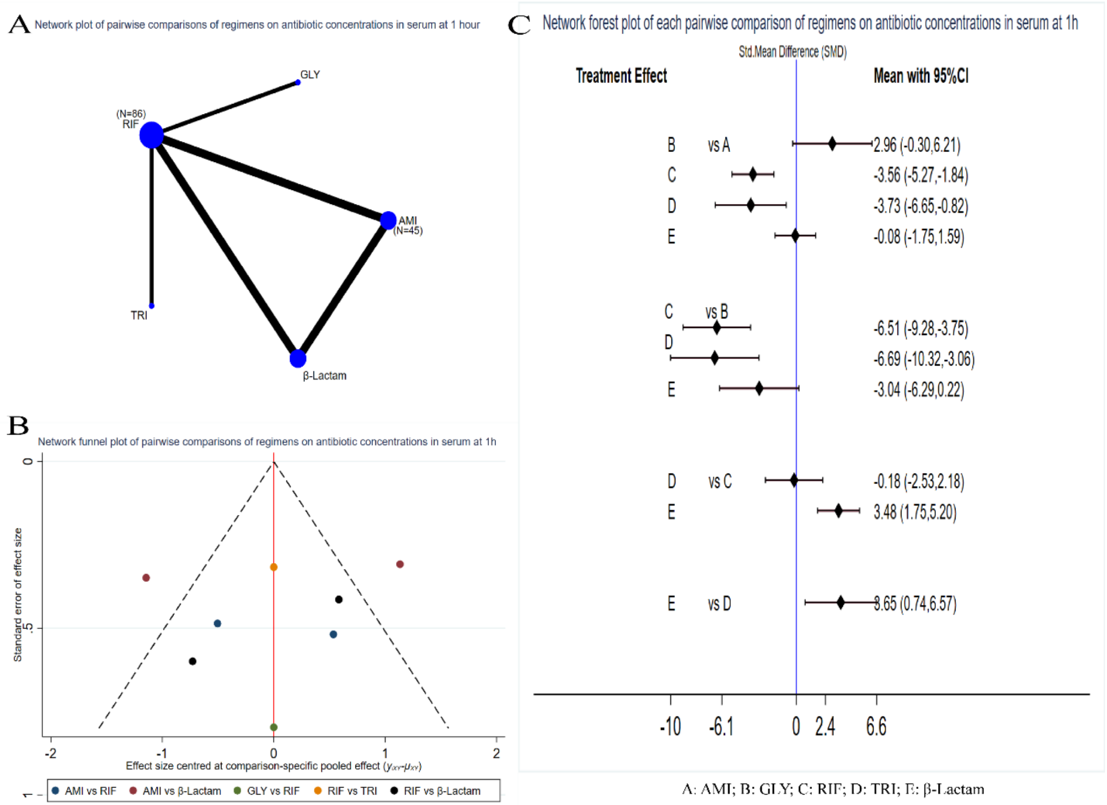

Supplementary Figure S1. Network Meta-analysis of antibiotic concentrations in serum at 1h after administration **(A)** Network graph; **(B)** The network funnel plot; **(C)** The network forest plot.

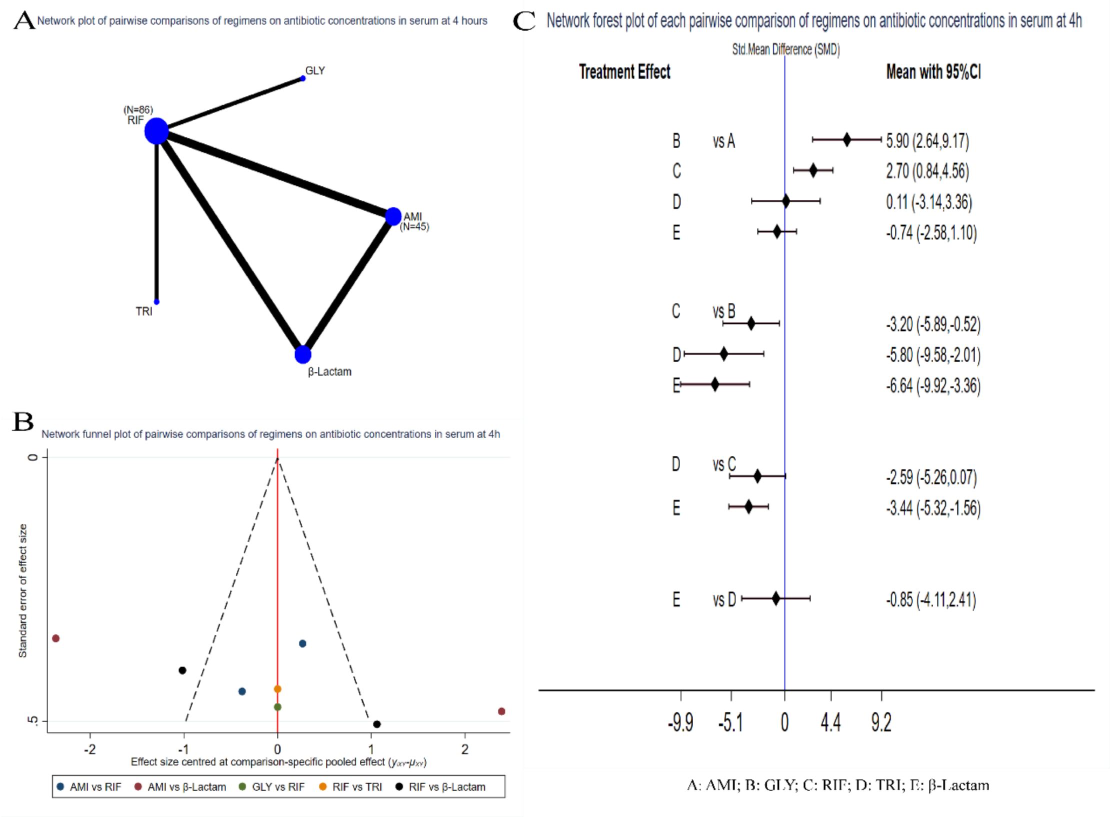

Supplementary Figure S2. Network Meta-analysis of antibiotic concentrations in serum at 4h after administration **(A)** Network graph; **(B)** The network funnel plot; **(C)** The network forest plot.

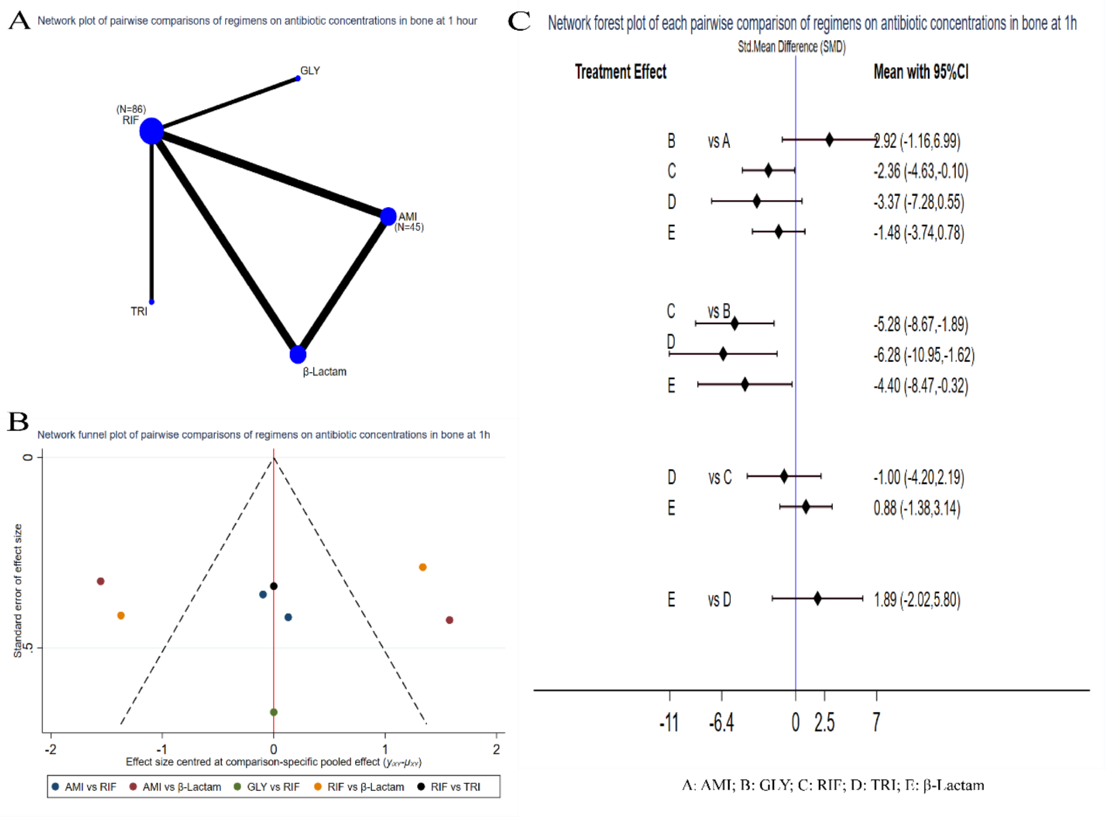

Supplementary Figure S3. Network Meta-analysis of antibiotic concentrations in bone at 1h after administration **(A)** Network graph; **(B)** The network funnel plot; **(C)** The network forest plot.

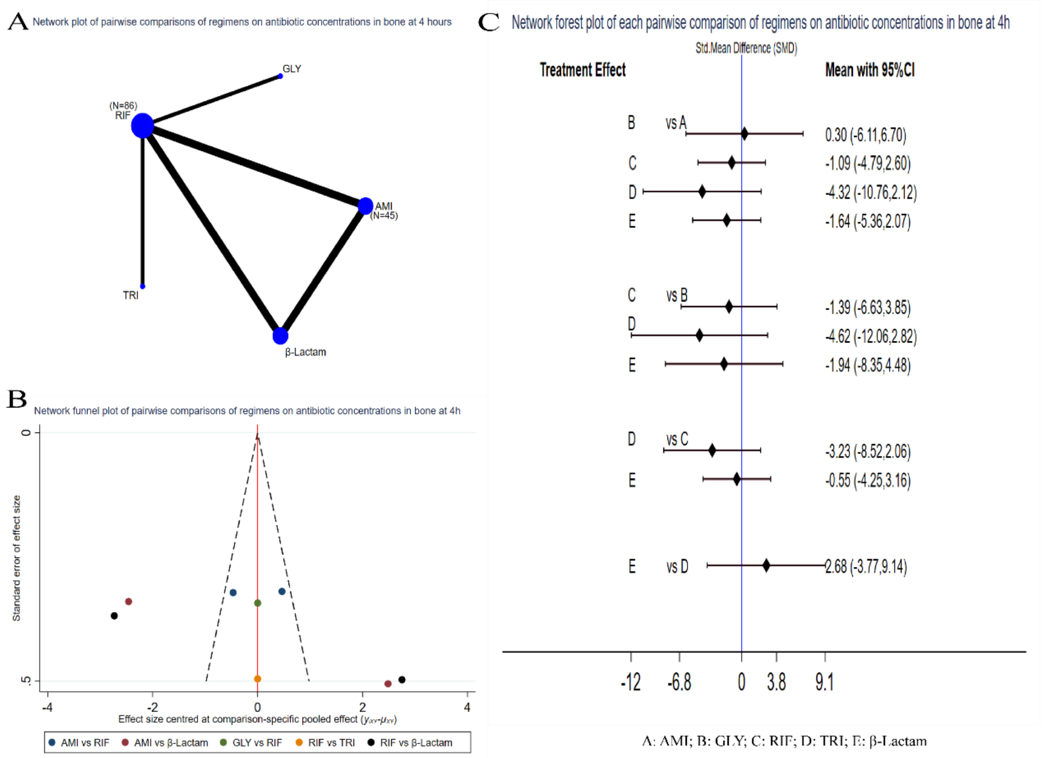

Supplementary Figure S4. Network Meta-analysis of antibiotic concentrations in bone at 4h after administration **(A)** Network graph; **(B)** The network funnel plot; **(C)** The network forest plot.
